# Well-Being and Pluralism

**DOI:** 10.1007/s10902-020-00323-8

**Published:** 2020-10-22

**Authors:** Polly Mitchell, Anna Alexandrova

**Affiliations:** 1 School of Education, Communication and Society, King’s College London, London, UK; 2 Department of History and Philosophy of Science, University of Cambridge, Cambridge, UK

**Keywords:** Well-being, Pluralism, Measurement, Methodology, Philosophy

## Abstract

It is a commonly expressed sentiment that the science and philosophy of well-being would do well to learn from each other. Typically such calls identify mistakes and bad practices on both sides that would be remedied if scientists picked the right bit of philosophy and philosophers picked the right bit of science. We argue that the differences between philosophers and scientists thinking about well-being are more difficult to reconcile than such calls suggest, and that pluralism is central to this task. Pluralism is a stance that explicitly drives towards accommodating and nurturing the richness and diversity of well-being, both as a concept and as an object of inquiry. We show that well-being science manifests a contingent pluralism at the level of methodology, whereas philosophy of well-being has largely rejected pluralism at the conceptual level. Recently, things have begun to change. Within philosophy, conceptual monism is under attack. But so is methodological pluralism within science. We welcome the first development, and bemoan the second. We argue that a joined-up philosophy and science of well-being should recognise the virtues of both conceptual and methodological pluralism. Philosophers should embrace the methodological justification of pluralism that can be found in the well-being sciences, and scientists should embrace the conceptual reasons to be pluralist that can be found in philosophical debate.

## Introduction

The idea that the science and philosophy of well-being would do well to learn from each other is well rehearsed. Recent calls for more interaction between the two fields emphasise the inevitable normative assumptions involved in defining and measuring well-being in the well-being sciences, and the dependence of philosophical theories of well-being on empirical facts about ordinary linguistic usage and psychology (Angner 2013; Bishop 2015; Prinzing 2020). These calls list mistakes and bad practices on both sides that would be remedied if scientists picked the right bit of philosophy and philosophers picked the right bit of science. Although welcome, this does not get to the heart of the issue, because the differences between philosophers and scientists thinking about well-being run deeper.

In this paper, we argue that a genuinely joined-up philosophy and science of well-being needs to embrace pluralism. Tendencies towards conceptual and methodological monism in well-being scholarship, which are clearly visible in the rising prominence and application of the construct of life-satisfaction, are, we argue, worrying and misguided. Our concerns are not merely academic—constructs and measures of well-being are increasingly used as decision-making tools in healthcare and public policy, and the consequences of using tools which fail to take seriously the conceptual complexity and epistemic uncertainty of well-being and its measurement have the potential to be widespread and grave.

Section 2 illustrates monism, first in science and then in philosophy. Contemporary well-being sciences adopt many different and often conflicting ways of defining and measuring well-being and in this sense manifest methodological pluralism. However, the landscape is changing. Within science, certain approaches to well-being, most notably life satisfaction, are starting to dominate the field. The enticing practical appeal of such approaches—which produce easy to work with quantitative data—threatens the prevailing, but fragile, pluralism. Philosophers of well-being, on the other hand, have tended to adopt a principled, anti-pluralistic stance in a search for the one true theory or definition of well-being. This monism in philosophy is, however, under attack, with defences of conceptually pluralist theories of well-being offered in its place.

We welcome the second development but condemn the first. Section 3 formulates distinct versions of pluralism as they would look given the aims of science and of philosophy respectively. Monistic approaches are tempting for their simplicity and usability, but their prevalence will ultimately undermine the goals of well-being sciences. We argue that this field would do well to preserve and to nurture a pluralist outlook. There are also good conceptual reasons for philosophers of well-being to adopt a pluralist stance. Section 4 defends each version of pluralism by showing that pressures towards monism harm epistemic goals in both science and philosophy. A joined-up philosophy and science of well-being should recognise the virtues of both methodological and conceptual pluralism. Philosophers should embrace the methodological justification of pluralism that can be found in well-being science, and scientists should embrace the conceptual reasons to be pluralist that can be found in philosophy.

## Monism Today

We use the term ‘monism’ to pick out ideas that reject one or another aspect of pluralism. In this section we show these ideas at work in empirical well-being research and then in philosophy.

### Monism in Science: A Fragile Pluralism Under Pressure

The empirical science of well-being is an interdisciplinary and multilevel field, spanning psychology, economics, sociology, geography and anthropology. So contemporary methods and approaches to well-being reflect the diversity of disciplines in social sciences. Its methods include experiments, surveys, ethnographies, interviews, and statistical analysis. This diversity is also visible in the many different definitions of well-being different researchers adopt and different measures that they devise to capture these definitions. Table 1 summarises what we see as the main traditions:

**Table 1:** Definitions and measures of well-being in current social and medical sciences

Definition	Measure
Happiness	Experience sampling, U-index, positive and negative affect scale, Spane, subjective happiness scale, affect intensity measures
Life satisfaction	Satisfaction with life scale, Cantril ladder, domain satisfaction
Flourishing	PERMA, psychological well-being index, flourishing scale, Warwick and Edinburgh mental wellbeing scale
Preference satisfaction	GDP, GNP, household income and consumption, stated satisfaction surveys
Quality of life	Human development index (capabilities), UK's office of national statistics measure of national well-being, Legatum prosperity index, social progress index, OECD better life index, Nottingham health profile, sickness impact profile, world health organization quality of life, health-related quality of life

This table is an abbreviated version. For a full version, references, and explanation of each row see Alexandrova 2017

As the table suggests, there is no single agreed way to define or measure well-being. Depending on the project and the disciplinary background of researchers, different metrics will be adopted. This diversity is amplified when we consider qualitative approaches to well-being practiced by anthropologists, some sociologists, journalists, and more generally writers of non-fiction narratives (Camfield et al. 2009; Steinberg 2015). These scholars do not typically bring a specific construct of well-being to their projects but instead they reconstruct it in conversation with their subjects. Using participant observation and extended interviews they unearth the local, culturally specific, and even individual categories that characterise good life for people they study. In recognition of these ‘manifest’ well-being constructs, we might add an extra row to Table 1:

The above discussion attributes to the science of well-being a diversity that we shall dub ‘methodological pluralism’. Such a pluralism is reflected in the large choice of measures and definitions summarized in the Tables 1 and 2. We use the wider adjective ‘methodological’ to emphasise the fact that adoption of a particular definition of well-being often brings a distinctive methodology, as, for instance, the adoption of a hedonist definition creates the expectation of quantitative measurement of psychological states. In attributing methodological pluralism to science we do not mean to suggest that scientists actually consciously and intentionally endorse this state of affairs. In fact individual scientists typically specialise in a single approach to well-being and sometimes even deny the value of the alternatives. Rather methodological pluralism is a macro-property of the field as a whole, stemming from its diverse disciplinary roots. But this pluralism is fragile and under pressure.

**Table 2:** An additional row to Table 1

Definition	Measure
Manifest well-being	Conversation, participant observation, interviews

Methodological pluralism is fragile in that it demands a lot from inquirers and there is an increasing pressure to scrap it. It demands in particular that researchers thoughtfully and consciously identify with a specific tradition and maintain awareness of other traditions they could be working in. This explicitly philosophical task does not come easily to scientists. So losing some of this diversity would undoubtedly make their work simpler. Indeed, there are powerful pressures from outside and from within science, which push scientists to seek greater unity.

Pressures towards monism can be identified at three levels: a pressure towards a single construct, a pressure towards a single measure of this construct, and finally a pressure towards a commonly pursued method.

Why should all researchers adopt the same construct of well-being? On the face of it, it makes little sense to insist that one construct of well-being is superior to all others. After all, happiness is one thing (an emotional state), broader quality of life is quite another, and claiming that the construct of happiness is superior to that of quality of life seems to commit a simple category mistake: they are distinct phenomena, each worthy of attention. Nevertheless, one construct enjoys far greater popularity and uptake, than others: life satisfaction. Life satisfaction is an attitude towards one's life as a whole and it summarises and aggregates attitudes about different spheres of life. In this way it lets people define their well-being however they prefer, incorporating whatever considerations they think are relevant.^1^ When attempting to justify this construct, researchers appeal to its simplicity and democratic nature (Diener et al. 2009, p. 47; Layard 2012; Clark et al. 2018, p. 4). Life satisfaction is also psychologically accessible: when people are asked how satisfied they are with their life as a whole they have no trouble answering this question (Frijters et al. 2019).

There is considerable debate about the validity of life satisfaction. Critics challenge its psychological, normative and psychometric presuppositions—life satisfaction assumes that people are able to aggregate a great deal of information about their lives, that this aggregation reflects their well-being rather than their culture, and that the data show appropriate psychometric properties.^2^ Despite the long history of these criticisms, life satisfaction has shown remarkable tenacity. In our view, this is due to the practical advantages of the approach. Life satisfaction is often represented just by one statement such as ‘I am satisfied with my life as a whole’ which respondents are invited to agree or disagree using a standard Likert scale. This single item appears alongside many other questions in big surveys such as the Gallup World Poll, the World Happiness Report, WHO's Health for All, and many other national and international datasets. Compared to other much longer questionnaires, these indicators are simply too good to pass up from the point of view of busy researchers pressed for time and resources. They are straightforward to implement, and generate interesting data that can be published and publicised beyond academia. Moreover, they are widely used, creating opportunities for cross study comparison and data linkage. In any case, none of the intellectual objections raised against these indicators have been able to dislodge them from their dominant status.

The strengths and popularity of life satisfaction indicators enable a more deliberate well-being monism. If there is a simple unidimensional measure of well-being, then it is possible to build a whole science around it—a science of the causes and consequences of this quantity. This is the idea behind a common approach to happiness economics. On its standard definition expressed by Carol Graham in the influential New Palgrave Dictionary of Economics, the goal of this field is to estimate an equation where subjective well-being is a quantity on the left-hand side and the vector of its causes are on the right-hand side:

Micro-econometric happiness equations have the standard form: *W*_*it*_ = *α* + *βx*_*it*_ + *ε*_*it*_, where W is the reported well-being of individual *i* at time *t*, and *X* is a vector of known variables including socio-demographic and socioeconomic characteristics. Unobserved characteristics and measurement errors are captured in the error term. (Graham 2008).

This vision of the science of well-being fits well with life satisfaction indicators. Although nothing in this picture logically implies that W needs to be life satisfaction rather than some other indicator of well-being, the left-hand side requires a single quantity and, apart from GDP—which the science of well-being is trying to unseat—life satisfaction is the only practical game in town.

Graham's vision has been taken up by numerous psychologists and economists. *The Origins of Happiness*—a recent book authored by a team of economists who represent the Wellbeing Programme of the LSE's Centre for Economic Performance—is a case in point (Clark et al. 2018). The authors defend a single, purely quantitative methodology, using data from various national and international panel datasets to make fairly precise inferences (and even causal inferences) about how much a given set of social, demographic, and economic circumstances boosts or impedes life satisfaction. Clark et al. carefully compile tables of coefficients designed to demonstrate, to the extent that the evidence permits, the causal strength of each factor that personal choices or policy can control. These coefficients become the evidence base for decisions big and small. Recently, this framework was used to evaluate the costs and benefits of releasing the Covid-19 ‘lockdown’ in the UK (Layard et al. 2020).

We present this vision as an example of a monistic trend because there is an ambition to find a single quantitative function (perhaps with coefficients adjusted to different conditions, but still one functional form) that represents the causal structure of well-being to be estimated using statistical and experimental data. This trend is motivated, though rarely explicitly, by a vision of a neat and internally consistent scientific paradigm in which all scientists work to further a single overarching goal using an agreed set of constructs and measures. In addition to this vision, monism has strong pragmatic dimensions. It endeavours to work with whatever is available and in whatever way that makes it easier to communicate to policy makers and other audiences. This monism seeks practicality, coherence, maximal coordination between different research projects with an eye on policy relevance.^3^ This is in contrast, as we shall see, to philosophical monism, which is more explicitly motivated by conceptual tidiness.

### Monism in Philosophy

The study of well-being in philosophy looks different from the sciences. Rather than studying determinants of well-being in particular social contexts, philosophers seek a definition of well-being that picks out all and only instances thereof. Well-being, in philosophy, is typically a general, all-things-considered assessment of how things are going for a person. There are some notable exceptions to this tendency—philosophers who recognise that well-being can be considered from different perspectives, from which different assessments might be appropriately made (Griffin 1988; Scanlon 1998; Kagan 1994; Tiberius 2011). But the prevailing orthodoxy in philosophy is to find the essence of well-being, and so to give a single, general answer to the question ‘What is well-being?’.

Of course, different philosophers give different answers to this question, with different theories grounding well-being in different properties: pleasurable affect, fulfillment of desires, or partaking in core human activities.^4^ But all these answers are monist in a central sense: they take ‘well-being’ to pick out some unique, determinate and identifiable property or state of people or of the world, which remains constant across all contexts. This entails that when we correctly identify well-being, we are always picking out the same thing. Typically well-being is taken to be what is good for a person all things considered, taking into account life as a whole. Some philosophical accounts are monist in a further sense, insofar as the state of well-being is always realised in much the same way or by much the same properties or attitudes (Feldman 2002). Others allow that well-being can be realised in a number of different ways: the specific things which make it such that someone has high levels of well-being differ from person to person, context to context (Fletcher 2013). Such accounts nonetheless take it that the thing that is picked out by the concept of well-being remains constant across people and contexts, even if the particulars which constitute it do not.

We propose to characterise well-being monism in philosophy with three commitments:

- (1) There is an essence of well-being;
- (2) The concept of well-being is clear and circumscribed;
- (3) Any theory of well-being must be general.

These commitments are not often explicitly acknowledged by philosophers who develop and hold monist theories of well-being but, we maintain, they nonetheless underpin most contemporary accounts. The three commitments are inter-related and should not be thought to be totally distinct from one another: the reasons for accepting one may also turn out to be reasons for accepting the others. We explore each in turn.

The first commitment is essentialism, which takes well-being to be a singular, identifiable and characterisable state or process that is out there in people or in the world to be identified and described. Essentialism implies that well-being always picks out the same thing at a sufficiently high level of abstraction. We can thus, in theory at least, identify an essence that characterises all and only instances of well-being, and so provide a clear definition of well-being which applies exhaustively across all contexts. Such an essentialism is likely grounded in a more general conceptual essentialism: the view that concepts (or at least some concepts) pick out a particular characteristic or set of characteristics which objects or states of affairs can have or fail to have (Wilson 2006). Concepts, on this view, are largely constant across time, and have a fixed and relatively determinate scope, meaning that applying concepts involves apprehending the concept and the objects under consideration, and determining whether the objects satisfy its requirements.

Secondly, well-being monists have strong circumscriptionist intuitions. This means that they will challenge any instance of usage of ‘well-being’ and related locutions which diverges from the sense familiar to philosophers (Alexandrova 2017; Mitchell 2018). The philosophical definitions of well-being described in this section clearly do not exhaust the ways in which ‘well-being’ is invoked in ordinary and technical language. Pace hedonists, ‘well-being’ is sometimes—often, even—ascribed on the basis of things other than pleasure or positive subjective psychological states, and sometimes denied despite the presence thereof. Philosophers who assert monist theories of well-being thus police the boundaries of the concept of well-being, circumscribing the ‘correct’ concept and ruling out alternative usage. Whenever there are challenges to the definition of well-being, or instances of well-being are implied or suggested which do not fit into a given definition, monist philosophers of well-being will endeavour explain why they in fact fail to pick out well-being, and perhaps instead pick out related or similar concepts (Hawkins 2019). This boundary policing will require an error theory to explain why language users may be mistaken about the meaning and reference of ‘well-being.’ Circumscriptionist intuitions are clearly closely related to the essentialism of monist theories: it is because well-being always picks out some unique property or state that alternative characterisations of well-being, which appear to pick out different properties, must be precluded. Essentialism will not brook much variety in the conceptual diversity of well-being.

Thirdly, monist philosophers of well-being are committed to the generality of their theories. This captures the idea that, rather than just considering some aspect or area of life, any theory of well-being must be formulated at a maximally general level. Different people leading radically different lives may be pursuing legitimately different visions of well-being but a single general theory identifies the key features of it. This preference for generality is, perhaps, more a matter of plausibility than a deep requirement of monism: if well-being picks out a single unique and characterisable property of things, it would be peculiar and highly improbable for it only to relate to some specific area of or time in people's lives—for well-being to be an assessment of people's physical health, or an assessment which occurs in childhood or at the end of life. For one thing, the less general the monist definition of well-being, the more difficult it will be to come up with a convincing error theory about mistaken ascriptions of well-being.

### Theoretical Friction

We have argued that there are monistic ideals in both science and philosophy. In science, monism seeks a single construct and a single measure of well-being, aiming to estimate a single well-being function to which all research should contribute. In philosophy, monism means a pursuit of a single general concept and theory of well-being. There are obviously important differences between these stances. Much research in the well-being sciences does not purport to have found the single, correct concept and theory of well-being. Incentives in sciences are different from philosophy and scientists spend less time insisting on the correctness of their paradigm over all overs and more time on putting it to work. When scientists show a measure of well-being to be valid, this does not automatically invalidate other measures. Monism in philosophy, on the other hand, is the orthodoxy to which there are a few challenges (as we shall see shortly) and the modus operandi is more explicitly confrontational: defending your own concept and theory of well-being means attacking others. Monism in science is motivated by practical considerations of gaining as much public influence for this research and simplifications as are worth the cost. Monism in philosophy is grounded in conceptual commitments and the desire for neatness and clarity.

The differences between the science and philosophy of well-being suggest some complications in the two fields using each others' findings to enrich their own work. To some extent it is possible to map constructs of well-being between philosophy and science: hedonic, eudaimonistic, and objective well-being constructs, for instance, can be found in both domains. But for monistic philosophers to justify their monistic theoretical claims using evidence from the well-being sciences risks overplaying the generalisability of such findings. For while evidence about the validity and nature of a hedonic well-being construct may lend support to a hedonic theory of well-being, it does not lend support to a *circumscribed, general, essentialist* hedonic theory of well-being. That is, it does not show that hedonic well-being is the single true theory of well-being, because the well-being sciences have produced a variety of validated, non-hedonic well-being constructs and measures. There is, perhaps, more scope for well-being scientists to use philosophical theories of well-being to underpin and justify their definitions and measures of well-being. However, if well-being scientists wish to maintain a broadly pluralist outlook about the conception and measurement of well-being—that is, if they see their measures and constructs as just a part of a bigger, pluralistic picture—they cannot use philosophical theories in their intended, monistic form. Of course the moves towards monism in well-being science open up the possibility of a more straightforward marriage of the science and philosophy of well-being. But the grounds for this depend on the justifiability of well-being monism, and of course the justifiability of the particular, monistic conception of well-being in question. Life satisfaction is not the preferred definition of well-being for most philosophers.

Well-being pluralism offers a means to reduce the theoretical friction between the science and philosophy of well-being. In the next section, we set out a positive vision of well-being pluralism in science and in philosophy, and suggest that there is reason to reject monism and accept pluralism in both domains. The ideal of pluralism will look different for sciences and for philosophy—we highlight methodological reasons for the well-being sciences to adopt a pluralistic outlook and conceptual reasons for the philosophy of well-being to do the same. In Sect. 4, however, we go on to argue that both spheres have a lot to learn from one another's reasons for pluralism.

## Pluralism

Pluralism is a stance that endorses the coexistence of two or more entities or processes, and denies that they can be reduced to one another or to some third entity or process. Well-being pluralism is, broadly, the idea that well-being is in some sense plural, and irreducibly so. To make this idea more informative we articulate what pluralism might look like, first, in the well-being sciences and, second, in philosophy. In both cases we suggest there are good reasons to endorse a form of well-being pluralism.

### Pluralism in Science

The idea that science can benefit from pluralism of methods, theories, or paradigms became commonplace in philosophy of science with the demise of ideals associated with logical positivism, most notably the unity of science. This ideal presumed that all worthwhile scientific theories will eventually be reduced to the fundamental physics and that all worthwhile scientific activities can be inscribed in a single universal method. These plans came under pressure when philosophers and historians of science showed a diversity in how scientists conceive of and practice their inquiry (Crombie 1995). Some went as far as arguing that theories with radically different ontologies co-exist with no prospect of unification (Dupré 1993; Cartwright 1999). On the tamer side, social epistemologists suggested that a community which pursues several approaches at once is better at generating healthy criticism and hence it is safer to maintain diverse scientific traditions in case the true one escapes us (Longino 1990).

‘The pluralist stance’ in science rejects ‘scientific monism’—that is, a search for a unified and complete account of the natural world using a single method of inquiry—and embraces ineliminable multiplicity of methods and theories, even if this comes at the expense of order and consistency (Kellert et al. 2006). Advocates of pluralism insist that the fact of plurality in science (a descriptive claim) motivates the attitude of respect for it (a normative claim). Recent defenders of scientific pluralism take the stance further and argue not only that multiplicity of theories and methods should be respected and monistic ideals abandoned; but moreover that we should foster and nurture a plurality of ‘systems of practices’ across sciences, actively encouraging parallel development of different scientific cultures, rather than merely allowing them to co-exist (Chang 2012).

In Sect. 2.1 we saw that the current landscape in the sciences of well-being is characterised by fragile pluralism with pressures towards monism. Are there reasons, motivated by these ideas from philosophy of science, to buttress this fragile pluralism and to resist the pressures for monism? Yes, we argue, first for measurement and second for methodology.

#### Measurement pluralism

Tables 1 and 2 illustrate the most visible pluralistic aspect of existing research. Researchers interested in keeping track of well-being face a wide array of choices. The first choice is to settle on the relevant construct. As a concept and as a locution, ‘well-being’ does not force on us any one particular option in the first column. We defend this idea more fully in Sect. 3.2 where we discuss conceptual pluralism, but for now let us work with the simple and uncontroversial idea that the sheer number of constructs of well-being shows that the field implicitly recognises legitimate diversity in definitions of well-being. This is in keeping with measurement in social sciences in general: the concepts of interest are often vague and fuzzy and yet the social scientists cannot, on pain of irrelevance, replace these concepts with well-behaved technical terms (Chang and Cartwright 2013). If so, care must be taken when any specific sense of well-being is used as a construct in a scientific project. Responsible researchers dedicate special attention to articulating the scope of the concept they take to represent well-being for their particular purposes. They do so in the methodology or introduction sections of research articles when they commit to treating well-being as, say, encompassing subjective evaluation of all the relevant aspects of people's life, or just an evaluation of their health state.

Pluralism about constructs of well-being amounts to the idea that the choice of construct when studying well-being is not obvious, that there are typically several options open to researchers, and that it is better not to eliminate these options. The science of well-being is not improved, the pluralist insists, by somehow reducing all the options in the first columns of Tables 1 and 2 to one. This is the first pluralist commitment.

The second commitment shifts attention to the second column of Tables 1 and 2, which summarise the measurement options corresponding to each construct. Even once the construct has been chosen, there usually remain several options about how it should be measured. Researchers must make a choice between measures that are self-reported and objective, between questionnaires and interviews, and about summary and multi-dimensional indicators. Once again, there are good reasons here to infer from plurality to pluralism. No questionnaire of well-being is superior to all others in every respect. Existing validation procedures are able to, at best, weed out measures that are obviously low in reliability and other indicators of validity. However they are not able to show obvious and uncontroversial superiority of any particular instrument.

There are several reasons for this. First, metrology, at least in the social and medical sciences, operates with several distinct facets of validity—content, face, construct, criterion, predictive, etc. Even though there are efforts to unify them, it always takes a judgment call to decide which of these to prioritise as evidence (Messick 1995). A given questionnaire may perform better on some subset of these criteria than on another subset. When this happens, considerations of content or face validity can pull against those of construct or predictive validities (for example, when an item of a questionnaire is important on theoretical grounds but fails to correlate with other items). There is no universal recipe for resolving these tensions and different researchers can legitimately arrive at different decisions. Secondly, construct validity—the most popular criterion—requires that the measure in question correlates with other measures that, according to our background knowledge about the construct, it should correlate with and does not correlate with those that it shouldn't. In this case, validation involves constructing a coherence framework of indicators and measures relating to well-being (Alexandrova 2017). In the absence of criterion validity, there is no gold standard against which a measure is tested to determine whether the thing that it measures is well-being or something else—rather, part of the assessment that it represents well-being is its coherence with other agreed measures and indicators of well-being. The way that different constructs, and different measures of those constructs, are understood to relate to one another takes something close to the form of a family resemblance concept. There is no single characteristic which is definitive of a valid measure of well-being; what determines whether something is a valid measure is how it hangs together with other measures of the same construct. Plurality of constructs and measures is thus both a legitimate outcome and a precondition of improvement of measurement.

Still, it is no secret that the actual choice of scales is often made on pragmatic grounds of ease of access, availability, tractability, and even just familiarity with the questionnaire. Researchers' time and resources are limited and it is unsurprising that these pressures will play a role. As a result certain focal points emerge. We have seen in Sect. 2.1 that there are monistic tendencies in this direction and that some small number of indicators emerge as winners. In happiness economics this is often life satisfaction, while in the health sciences it is health state utility as measured by EQ-5D. But while there are clear winners in such popularity contests, they are certainly not uncontroversial when it comes to validity. As we already mentioned, life satisfaction does not track emotional well-being, can be vulnerable to context effects, and makes implausible psychological assumptions about judgement and perception. Its defenders also claim that it is the most democratic and liberal measure, allowing people to fill in their own values, rather than using the values of the researchers who, if using multiple indicators, have to exercise a value judgment when deciding how to weigh them (Clark et al. 2018; Layard 2019). But this is a very limited sense of ‘democratic’, since when people are asked to rate their life satisfaction there is no genuine conversation of what well-being means to them and there is no option for the subjects to reject the very idea that life is a good with which one can be satisfied or dissatisfied as with a product. Some evidence about folk conceptions of well-being suggests that well-being is not understood to be purely subjective (Phillips et al. 2011, 2017; Kneer and Haybron 2020).

EQ-5D, which was developed not as a comprehensive instrument, but as a complement to other quality of life measures (Brooks and Group 1996), has limitations of its own. The single aggregate values assigned to health states mask substantial heterogeneity in respondents preference ordering, suggesting that a large proportion of people would disagree with the overall ordering of health states (Roberts and Dolan 2004). Moreover, there is evidence that EQ-5D performs less well as a measure of quality of life with respect to mental disorders than physical disorders (Brazier 2010). Such findings suggest that EQ-5D should be used with caution, and that there are limitations to its appropriateness as a measure of quality of life. Regardless, EQ-5D is overwhelmingly the most popular multi-attribute health state utility instrument (Richardson et al. 2011; Wisløff et al. 2014).

These criticisms do not diminish the popularity of these measures, but—as suggested in Sect. 2.1—we strongly suspect this is not because researchers fail to recognise these problems or have convincing answers to them but rather because they are seductively easy to work with: they are widely available through panel datasets and generate a single number that can be straightforwardly plugged in into statistical analysis. An additional advantage of these popular measures of well-being comes from their very popularity: the more researchers use them, the easier it becomes to compare their data, and the simpler it becomes to establish and maintain fruitful scientific communication.

Simplicity, convenience, and entrenchedness of life satisfaction or EQ5D are genuine considerations in their favour. It is certainly better to have a science of well-being based on them only than no science at all. It is also desirable to have a science that responds quickly to new social priorities and, since life satisfaction or EQ5D data are more easily available than other data, it is plausible that these indicators be prioritised by researchers. However, these pragmatic considerations should not be overstated. We do not typically face a choice between using life satisfaction only or else ignoring well-being entirely. A science of well-being based merely on data about satisfaction strikes us as hopelessly impoverished. It loses the richness of multidimensional indicators, of structured and unstructured interviews, and of questionnaires that gauge deeper states such as happiness and flourishing. It forsakes the duty, widely acknowledged in this field, to reflect the diversity of human experience (Stiglitz et al. 2010).

To sum up, measurement pluralism seeks to preserve the variety of well-being constructs and measures, as a way of respecting the complexity of well-being and acknowledging uncertainty in its measurement and validation. Next we highlight a further pluralist commitment.

#### Methodological Pluralism

While measurement pluralism concentrates on the choice of indicators to gauge the relevant construct of well-being, methodological pluralism is broader, encompassing not only what data is collected but also how widely it is collected, how it is analysed and what conclusions are drawn from it. A methodology is pluralist in this sense when it is based on a recognition that in each of these aspects researchers have several plausible options and that using more than one approach has distinctive advantages. To make these ideas less vague it is worth returning to an example of monist methodology from Sect. 2.1.

Happiness economics as a field takes as its goal the formulation and estimation of an equation on the left-hand side of which is the quantity represented by life satisfaction and on the right-hand side is the vector of demographic and socioeconomic variables plucked from available statistics. Our concern is how much this methodology conceals. We have already seen the problems with relying solely on life satisfaction as a measure of well-being, but, in addition to this, the very idea that knowledge of well-being amounts to estimating an equation is limited and shallow. The first problem is that the variables in the equations are not derived from the best available theories about determinants of well-being. Housing, unemployment, poverty, and other official statistics, may be convenient and interesting, but if the project is to understand causes of well-being, then we need to look deeper and wider. We need models of well-being at different scales of analysis recognising the phenomenon at psychological, cultural, and social levels. The second problem is that the coefficients attached to the variables (even when there are sufficiently fine-grained and valid data to estimate them) do not reveal the underlying mechanisms behind the correlations. The econometric analysis on its own is insufficient to address these problems and this is where qualitative or mixed-methods are necessary. Researchers in these traditions have the tools for uncovering phenomena that are often invisible to available statistics. Through interviews and fieldwork they reveal the lives and stories behind the correlations and this knowledge is sorely needed to improve both definition of variables and the understanding of mechanisms (Singh and Alexandrova 2020; Illari 2011).

The above is a case for combining methods, but another form of methodological pluralism is to foster research programs based on distinct philosophical assumptions. For instance, pluralist considerations would support enabling the parallel running of traditional happiness economics, alongside happiness sociology, happiness anthropology, happiness psychology, or any combination of these, and so on. Just as there is no pragmatic case for reducing all constructs and measures of well-being to one, there is no pragmatic case for organising the science of well-being around any particular model, whether from economics or from psychology. Compromises between validity and practicality are real and legitimate. But if these compromises mean that well-being research has to fit one mould, this is too high of a price to pay.

To take stock, pluralism about the science of well-being is committed to diversity of constructs, measures, and methods. It need not endorse the stronger forms of pluralism that appeal to fundamental disunity of nature. Rather pluralists recognise distinct epistemic cultures around well-being and cherish them because they reveal different aspects of well-being and because there is much uncertainty about which is the most fruitful.

### Pluralism in Philosophy

In this section we set out two ways in which a philosophy of well-being can be pluralist. The first we call constitutive pluralism. As briefly mentioned in Sect. 2, many monist theories of well-being are pluralist in an attenuated sense: while well-being refers to a particular, identifiable state, it can be constituted by several different properties or objects. The second we call conceptual pluralism. This is a more radical form of pluralism, which is inconsistent with the three commitments of philosophical well-being monism which we set out in Sect. 2.2. Conceptual pluralism about well-being entails that there is no single essence which characterises all and only instances of well-being. Instead, there are many different, inconsistent concepts of well-being, which are appropriately invoked in different contexts and at different times.

#### Constitutive Pluralism

Constitutive pluralism captures the idea that well-being is made up of multiple, irreducible components. There are a number of different ways in which well-being might be constitutively plural, and we suggest that most philosophers thinking about well-being are constitutive pluralists in some sense.

Objective goods theories of well-being typically take well-being to be constitutively plural, specifying a list of goods that contribute to well-being. Take, for example, Guy Fletcher's (2013) list of prudential goods: achievement, friendship, happiness, pleasure, self-respect, virtue. We are well off to the extent that we have these goods in our lives. Other goods can contribute to our well-being only insofar as they contribute to, or can be understood in terms of, a good on the list. These goods are not reducible to one another or to some master value, and they are collectively exhaustive of well-being. The ‘constitutive’ relationship between the goods on the list and well-being is non-instrumental. So the presence of the goods in someone's life doesn't *cause* them to have higher well-being, rather, their well-being is *constituted by* the goods: they do well because and to the extent that they have them in their life. Some hedonist accounts of well-being are constitutively plural insofar as they recognise a number of irreducible kinds of pleasure, any of which might at different times and contexts constitute well-being (Crisp 2006). So the pluralism of constitutive plurality might sometimes involve there being multiple irreducible varieties of the same kind of entity, and sometimes several kinds of entity.

Monistic accounts of well-being can struggle to capture the fact that our lives can go well and badly in radically different ways. Constitutively plural accounts attempt to capture this by setting out a range of contributors to well-being. While many constitutive pluralist accounts take well-being to be an overall assessment of a person's life as a whole, they can also recognise the role of context—allowing, for example, that the constitutive elements of well-being can vary for different people or at different times in a person's life. Constitutive pluralism is well represented in the well-being sciences. Many measures of well-being are multi-dimensional, distinguishing, for instance, between the emotional and cognitive aspects of well-being, or resisting converting the different dimensions of well-being to a single summary value.

Many philosophical theories of well-being adopt a constitutive pluralist approach because they recognise that monist accounts of well-being end up in sticky situations. Insisting that well-being is always and only constituted by one good (e.g. pleasure, desire, happiness) comes into conflict with core intuitions about well-being or linguistic applications of well-being. If well-being is always and only constituted by the satisfaction of our desires, then why don't we think that the heroin addict's desire for his next fix is good for him? If well-being is always and only constituted by our being happy, then why do we think that the pursuit of demanding, frustrating, laborious projects can be central to our well-being? Monistic views can insist that someone is doing well or badly when they clearly aren't, on the grounds that their lives do not contain a particular monistic prudential feature. The idea is, then, that there must be something other to well-being than these single goods if we are to make sense of the ordinary scope of the concept of well-being.^5^

As mentioned in Sect. 2.2, constitutive pluralism about well-being is compatible with a form of well-being monism. Constitutive pluralists about well-being need not suggest that well-being means different things in different contexts and for different people, but rather that well-being comprises a plurality of things. Something similar might be said about other concepts that are both descriptive and evaluative, like beauty, courage or justice. In all cases, there is something relatively determinate which these concepts pick out, but the properties through which the concept is manifested may be quite different across contexts. So constitutive pluralism about well-being is not necessarily anti-essentialist.

However, the constitutive pluralist does recognise a gap between the high-level definition of well-being and the ways in which it is realised and specified in particular contexts—even if there is an identifiable essence of well-being, this doesn't exhaust what can be said about well-being in different contexts, and we might need quite a number of different tools for identifying and measuring well-being. Recognising the complexity and diversity in the manifestation of well-being introduces some ambiguity in the application of the concept of well-being. In order to know whether someone is doing well or not, we need to know not just what the essence of well-being is, but whether that essence is realised by the constitutive elements of well-being in this instance. This puts some pressure on the essentialist commitment. The high-level definition of well-being which remains the same across contexts provides only some information about how to identify and comprehend well-being in particular cases. So understanding the concept of well-being in any given case involves contextual knowledge and interpretation as well as general theoretical knowledge.

#### Conceptual Pluralism

Conceptual pluralism is a more radical form of philosophical pluralism than constitutive pluralism. It entails that there are multiple competing concepts of well-being which are appropriately invoked in different contexts. Conceptual pluralism about well-being has been defended by philosophers under the banner of ‘contextualism.’ All versions of well-being contextualism share the core claim that the meaning of well-being is always indexed to a context (Darwall 2002; Campbell 2016; Alexandrova 2017; Mitchell 2018). Depending on facts about the person whose well-being is under consideration, the environment in which well-being is being assessed, and the purposes of the person or people making the assessment, different concepts of well-being are appropriate. For contextualists, the indicators that someone is doing well—the things that are relevant and irrelevant to well-being—and the thresholds for doing well and badly can differ across contexts. Different concepts of well-being might share some core common content or function but this commonality is at a high level of abstraction and doesn't exhaust the meaning of well-being. While different instances of well-being likely share some characteristics with one another, there is no definitive list of characteristics determinative of well-being. The overall concept of ‘well-being,’ for contextualists, is thus relatively thin.

On this view, finding a single essence of well-being which runs through all of these diverse conceptions is a fool's errand. While contextualists recognise that there are some global concepts of well-being—which, for example, seek to define a concept of well-being for human beings in general—these are not superior to more localised concepts on account of having a broader remit. In fact, like more localised concepts, global concepts are appropriate only for particular purposes. Contextualism about well-being denies that different concepts of well-being are merely fragments of an overarching concept, such that when concatenated they give us a full picture of a person's well-being. Many local concepts of well-being will be inappropriately applied to many people: concepts of child well-being, end-of-life well-being, disease-specific well-being, and so on, only make sense when used with particular people, in appropriate contexts.

The way that well-being is understood in the well-being sciences is suggestive of conceptual pluralism. Depending on whether well-being is being defined and measured by and for clinicians, psychologists, economists or public policy professionals, conceptual pluralists take it to consist in different factors and characteristics. The construct of well-being used by a palliative care nurse to assess the well-being of his patients may hardly overlap at all with the construct of well-being used by an urban planner to assess the impact of her plans and policies on citizens' well-being. To invoke factors from the urban planning construct in the palliative care context would be incongruous, perhaps even unintelligible, and wouldn't help to expand or improve the nurse's understanding of his patient's well-being. Even when their assessments incorporate general measures of well-being, these are likely to differ depending on contextual factors—the palliative care nurse might be more likely to use a general measure of subjective well-being or life satisfaction, whereas the urban planner might be better served by an objective measure, which takes into account access to parks, public services, sports facilities, and so on. Different contexts may, then, use genuinely localised as well as locally-appropriate general well-being constructs. Conceptual pluralism about well-being thus captures something of the conceptual variety that characterises technical and non-technical invocations of well-being.

Monistic and constitutive pluralist conceptions of well-being typically take the scope of the concept of well-being to be a person's whole life or their whole life at the time of consideration. That is, well-being for these philosophers is an assessment of how well someone's life is going all things considered. In the spirit of circumscription they deny that many localised concepts of well-being are definitions of well-being at all, instead capturing well-being but adjacent or related concepts, such as happiness, health, or psychological functioning. At best the localised concepts are understood to capture fragments of the concept of well-being. For the conceptual pluralist this is futile. The palliative care nurse doesn't know more about his rural-dwelling, retired patient's well-being if he also assesses her urban-development-related-well-being, her workplace-related-well-being, her neo-natal well-being, and so on. These other localised assessments of well-being are context inappropriate. Moreover, to draw categorical conceptual lines between well-being and closely related concepts fails to recognise that sometimes well-being is constituted, or partly constituted, by health, life satisfaction, pleasure, and so on, so this cluster of concepts are not just inter-related but inter-defined.

For the conceptual pluralist, to deny that many localised definitions of well-being are concepts of well-being at all, and to insist that only general whole-person or whole-life concepts capture well-being in its fullness artificially constrains the ordinary usage of the concept of well-being. To claim that a tool for measuring well-being is in fact a measure of happiness or of psychological functioning when it is designed and understood as a measure of well-being is to engage in a form of conceptual policing without a clear justification. It is not clear that philosophers should get the final say on what can be called ‘well-being’—though they can certainly add their own concepts of well-being to the mix and see whether anyone finds them plausible.

### The Global and the Local

So how radical is conceptual pluralism? In this section we take the opportunity to correct possible misconceptions about this view, in particular the worry that it might go too far in privileging local over global concepts of well-being.

If well-being pluralists deny that there is a single essence which runs through all conceptions of well-being, then it's reasonable to ask why we need to retain an overarching concept of ‘well-being’ at all. Perhaps it is content-free and needs to be eliminated. We do not take this route. Well-being pluralism is consistent with there being some very general shared content to all well-being uses—something relating, perhaps, to how someone is doing or faring. However, this content is too limited to specify the content of each contextual well-being ascription. Making an ascription of well-being requires substantive specification of a concept of well-being which sets out the conditions under which someone can be understood to be doing well or badly. And furthermore, the conceptual content shared by all uses of well-being is too thin to pick out only instances of well-being—how someone is doing or faring can refer to aspects of them or their life other than their well-being.

Many linguistic communities, notably the contemporary Anglophone one, do in fact use term ‘well-being’ to group together a number of different constructs. But whenever well-being is invoked or ascribed in practice, additional substantive conceptual content is appended to this thin concept of well-being to develop specific general or local concepts of well-being. Philosophers have attempted to flesh out an acontextual concept of well-being and to use it for various theoretical purposes. If, and to the extent to which, their concepts work for these purposes, we see no problem in retaining some overarching concept of well-being. But for practical purposes such acontextual concepts are simply not sufficient. They will not enable us to understand whether and when someone is doing well, and how well they are doing—and so will not allow us to pick out well-being and to make well-being ascriptions in practice.

Those with prudentially monistic inclinations might contend that an objective list theory of well-being makes better sense of the relationship between the central concept of well-being and contextual invocations and ascriptions of it. As we noted in Sect. 3.2.1, objective list theories are sensitive to a certain amount of contextual variation in the meaning and nature of well-being, whilst defending a relatively substantive conceptual core which is shared by all contextual variants. Many objective list theories of well-being specify a short, finite list of things which comprise well-being (knowledge, friendship, happiness, achievement, health, practical reason, self-respect). According to objective list theories of well-being, any ascription of well-being must reflect the goods on the list—which of them are present in someone's life, in what combination, and to what extent. For the objective list theorist, both the urban planner and the palliative care nurse will need to consider the presence of the same basic goods in the lives of their patients or the citizens affected by their work—their health, friendship, happiness, self respect, and so on—although these goods will be to some extent differently realised in each case.

According to our taxonomy, objective list theories are constitutively plural. But the discussion at the end of Sect. 3.2.1 suggests that there is actually relatively little space between constitutive pluralism and conceptual pluralism as we understand them. If the objective list theorist must specify the meaning and nature of each of the goods on the list in each context of application—what ‘friendship,’ ‘health,’ ‘happiness’ and so on mean for the palliative care patient and the citizen—then there remains significant substantive conceptual content in contextual well-being constructs which is not included in the overarching concept of well-being. The difference between this account of well-being and our conceptually pluralist approach might best be understood to come down to differences in how substantive the overarching concept of ‘well-being’ is—that which remains constant across contexts.

More obviously monistic objective list theories with short lists of goods that are narrowly defined and include strict rules of application are, we think, likely to be relatively unconvincing as theories of well-being. Inevitably some degree of massage and manipulation will be required to explain how the same highly specified goods (exhaustively) comprise well-being in all contexts. More permissive objective list theories will, for example, allow goods to come under different descriptions in different contexts, take some goods to be irrelevant to well-being in certain circumstances, and adopt long or open ended lists of goods. While more likely to be able to accommodate enough flexibility to capture a wide variety of contextual well-being constructs, these start to look less monistic and more conceptually plural, insofar as the overarching well-being concept must become increasingly thin in order to harbour appropriate diversity.

More nuanced objective list theories include Guy Fletcher's defence of what he calls *aspectualism* about well-being—the view that in different contexts we are interested in different *aspects* of well-being (Fletcher 2019). General assessments of well-being, for Fletcher, capture well-being proper, and will consider a person's overall well-being, taking into account particular aspectual assessments. Amartya Sen takes well-being to consist in the achievement of functionings, that is, activities and states of existence which capture how a person can function (Sen 1985). For Sen, prudentially relevant functionings comprise a large and unconstrained set, which can be broad or narrow and can come under overlapping descriptions. On his account, different sets of functionings, under different descriptions, will be relevant for well-being in different contexts. The overarching concept of well-being in such accounts is very thin. It can perhaps be best understood as a conjunction or disjunction of different contextual well-being constructs (some of which are very localised and some of which more general) rather than adding much or any substantive conceptual content. When the list of potential contextual well-being constructs is open ended, it's difficult to see how this differs meaningfully from our pluralist account.

In summary, we do not deny or eliminate the overarching concept of well-being. Rather we maintain that, to the extent that this exists, it is conceptually thin and needs substantive specification in order for it to be used to make well-being ascriptions in practice. We understand there to be relatively little space between our conceptual pluralism and sufficiently nuanced objective list theories. There seems to be little reason to insist that the latter are conceptually monist, as the extent to which they specify an essence which runs through all instances of well-being is extremely limited.

## Defending Pluralism

So far, we have seen that the applied sciences and philosophy approach the study of well-being very differently from one another. Their motivations for defining and, in the scientific case, for measuring well-being produce not only divergent definitions, but also different (often implicit) conceptions of the kind of thing that well-being is. The diverse, localised searches for context-specific measures of well-being in the sciences have led to contingent pluralism. The search for a single, essentialist definition of well-being by philosophers has led them to principled anti-pluralism about well-being. We have argued that, despite the pragmatic arguments for a move towards monism in the science of well-being, there is in fact a strong methodological basis for well-being pluralism. Moreover, despite the resolute monism of much philosophy of well-being, we have argued that there is a conceptual basis for well-being pluralism. In this section we go further, arguing that both the well-being sciences and the philosophy of well-being would benefit from recognising one another's reasons to be pluralist: philosophy should recognise the methodological reasons, and science the conceptual reasons, for well-being pluralism.

First, then, there is reason for the well-being sciences to recognise conceptual pluralism about well-being. In the previous section we suggested that the pragmatic considerations for moving towards well-being monism in the science of well-being are limited. Though there are potentially strong reasons of convenience for using the same measure across different contexts, and for preferring simple, easy-to-use measures, convenience only goes so far. In particular, reasons relating to convenience do not pass much muster when the validity of measures is questionable. Assessing construct validity, for example, requires prior knowledge of the construct in question—in this case, well-being. This prior knowledge can't just come from other validated measures of well-being, or at least, insofar as it does, these other measures must be grounded in some externally acquired knowledge about well-being. In other words, we need some prior, conceptually coherent account of what well-being is in order to know whether we are measuring it correctly (McClimans and Browne 2011; Alexandrova 2017).

Theoretical reasoning about well-being can provide at least part of this background knowledge. Philosophical concepts of well-being set out, in relatively broad terms, what kind of a thing well-being is and how to recognise it, offering a standard against which to assess the relevance of potential indicators of well-being. If we are right to think that there are strong reasons for philosophers to favour conceptual pluralism about well-being, then these reasons should be recognised as such by those situated within the well-being sciences who are seeking to develop valid measures of well-being. Of course, in one sense, recognition of the conceptual plurality of well-being makes the task of validating a well-being construct somewhat more difficult: if there are multiple different possible concepts of well-being at play, then there will not be a single, clear theoretical construct against which to compare measures. Conceptual pluralism about well-being implies that more theoretical work needs to be done upfront to determine the appropriate conception of well-being for the measurement context and well-being subjects under consideration. It also suggests that mere absence of strong correlation of one well-being measure with others need not undermine its validity. If there is good reason to think that they each measure different concepts of well-being, then they may diverge in core ways.

Secondly, there is reason for philosophers to take on board pluralism of the sciences. We have argued that there are good reasons to be a constitutive pluralist about well-being, which are accepted by many if not most philosophers thinking about well-being. However, we also suggested that constitutive pluralism opens up a gap between the high-level definition of well-being and its manifestation in particular contexts. Determining what well-being means in a particular context and assessing the level of someone's well-being involves reference to something other than the central definition of well-being. In particular it requires reference to the multiple possible kinds of goods that might be constitutive of well-being, and some assessment as to the presence or absence of these. In short, any contextual identification of well-being is going to require some kind of measurement. This might be measurement in a very limited sense—identifying the presence or absence of some object, for example, or ordering a set of actual and potential states without specifying any intervals between them—but it nonetheless involves more than merely theoretical, definitional work. This central position that measurement occupies in thinking philosophically about well-being suggests that philosophers must take the practice of measuring well-being seriously. And if, as we have argued, there are good reasons for adopting measurement and methodological pluralism about well-being, then these reasons also apply to philosophers trying to acquire knowledge of well-being. Of course, what such ‘epistemic’ pluralism about well-being means for philosophers is going to be slightly different in the details from what it means for well-being scientists—philosophers are not typically in the business of surveying people or collecting scientific data, for instance. For philosophers, epistemic pluralism might entail an openness to the different possible ways of characterising someone's life and its objects in order to make assessments of well-being, as well as consideration of the different perspectives from which such a characterisation might be made. Epistemic pluralism suggests that, even once a given well-being concept has been determined to be appropriate for a particular context, there will be different ways of applying it, which might lead to different contextual assessments of well-being.

So, both well-being scientists and philosophers should pay attention to the reasons each other have for being pluralists about well-being. This is, perhaps, unsurprising, especially if we conceive of well-being scientists and philosophers as trying—at some level—to do the same thing: to say something useful and true about what well-being is and how to come to know it. In the remainder of this section, we will suggest that there might be less distance between the principled and methodological reasons for being a pluralist about well-being than our arguments thus far have supposed, setting the stage for a more joined up science and philosophy of well-being.

A full-blooded conceptual pluralism, of the kind we set out in the previous section, goes hand-in-hand with a rejection of the essentialist theory of concepts with which we characterised philosophical well-being monism. In contrast to this essentialism—which sees concepts as relatively stable, determinate, and providing necessary and sufficient conditions for their own application—a non-essentialist account of concepts takes them to be much more indeterminate and requiring contextual specification in order to determine their scope and meaning (Wilson 2006). Concepts, on this non-essentialist view, don't take the form of clear and abstract definitions, but rather must be understood in relation to their application. And moreover, this application is typically messy, with appropriate usage of concepts changing over time and exhibiting variation over different linguistic and social contexts. On such a non-essentialist view of concepts, coming to understand the nature of a concept like well-being necessarily involves careful attention to the diversity of ways in which it is used and entreats us not to look upon variation in use as a concerning or unusual feature.

Part of the motivation for conceptual pluralism comes from a concern to take at face value the apparent conceptual variation in the way that well-being is used and ascribed. Of course, looking carefully at the way that the concept of well-being is used as a means of coming to understand the concept, and as a means of criticising essentialist definitions thereof, already calls into question the essentialist approach. So, the argument for conceptual pluralism should be seen as presenting an alternative to, rather than a knock-down critique of, the kind of philosophical monism that we set out in Sect. 2. But if taking seriously the actual scope and expression of well-being seems like a sensible approach to thinking about the meaning of the concept, then the wide variation exhibited both in ordinary language and technical contexts suggests that there are reasons to be conceptually pluralist about well-being. And these reasons are both principled and pragmatic in nature, because a non-essentialist account of concepts suggests that our theoretical development of concepts must look out towards actual usage in order for definitional content to be meaningful. This doesn't have to mean that attempts to define well-being can't engage in any circumscription of conceptual boundaries at all, nor that they can't be critical of particular usages of the concept. But it does suggest that such critique can't take place in abstraction from, and without paying close attention to, actual usage and its variation.

So what does this mean in practice for the philosophy and science of well-being? First, it suggests that pragmatic and methodological considerations about the meaning and measurement of well-being, such as the richness and diversity of its application, the variety of possible approaches to the characterisation and analysis of putative indicators of well-being, and epistemic uncertainty around making assessments of well-being should be central to conceptual theorising about well-being. These are features which come with the territory of seeking to define a complex, normative, social concept, not idiosyncrasies to be abstracted away. And it also suggests that the theoretical constructs that the well-being sciences draw on in order to develop and validate measures of well-being can't be generated entirely separately from the practice of using and thinking about well-being. This doesn't mean that the well-being sciences can do away with theoretical concepts of well-being: theoretical concepts of well-being don't just describe or report well-being usage, but interpret, characterise and generalise about it. This brings the science and philosophy of well-being much closer together and envisages them as­­ reflectively engaged in a shared practice of theorising about, describing and measuring well-being.

## Conclusion

The well-being sciences and the philosophy of well-being have historically approached the study of well-being very differently. These differences make it difficult for philosophers and scientists thinking about well-being to use one another's scholarship to inform or augment their own. Failure to recognise underlying conceptual differences between the two fields risks using philosophical arguments or scientific evidence about well-being in ways which do not adequately reflect their motivations or appropriate scope of their application. We have suggested that moving towards a considered pluralism in both domains will allow some of this tension to be resolved, and enable science and philosophy to integrate empirical and theoretical work in a constructive and conceptually coherent manner. The reward is not only inter-disciplinary progress. The reasons to be a well-being pluralist extend beyond pragmatic considerations and conceptual coherence. Well-being monism, whether as a methodological or theoretical commitment, fails to recognise the conceptual complexity of well-being and the substantial epistemic uncertainty surrounding its identification and measurement. There are independent methodological and conceptual reasons for endorsing well-being pluralism within both philosophy and the sciences.

Our defence of well-being pluralism is not merely an intellectual exercise; how well-being is understood and treated as an object of study has wide-ranging and hefty consequences. Since measures of well-being are increasingly used in healthcare and public policy, as a tool for distributing resources and aiding judgements which profoundly affect our individual and collective lives, the inclination towards monism in the well-being sciences becomes positively dangerous. The move towards fewer and simpler tools for the characterisation and measurement of well-being risks underestimating its complexity and variability. In relying on public authority of simple quantification, monism overstates the certainty of our knowledge. As the potential effects of mistaken, unfair or unrepresentative assessments of well-being grow more and more consequential, it is increasingly imperative to be sensitive to the dangers of over-simplification. We urge well-being scientists to avoid inadvertently endorsing a form of well-being monism by choosing well-being approaches and measures on tempting but ultimately limited pragmatic grounds. And we urge philosophers to connect their theoretical work about well-being to the urgent questions which are occasioned by the sciences and by the invocation of well-being in public policy and public life. Delivering theoretically and methodologically nuanced data and analysis to support these demanding public roles requires both scientists and philosophers, working together, to embrace a pluralistic outlook and to avoid the lure of pragmatic shortcuts and conceptual tidiness.

## Data Availability

There is no supporting data or material.

## Footnotes

1 This is also the justification given by defenders of life satisfaction in philosophy (Tiberius and Plakias 2010), though these commentators do not insist that life satisfaction is uniquely superior to all other constructs.

2 For psychological and normative critique see Haybron 2008; for ongoing worries about psychometric validity see Deaton and Stone 2016 and replies.

3 At a recent OECD conference on well-being, Richard Layard made the strong claim that: “Unless you've got a single concept against which you're judging everything, you can't claim to have a coherent set of policies” (Layard 2019). In this paper we will contest the idea that coherence requires monism—rather, we suggest, conceptual coherence points towards well-being pluralism.

4 See Crisp 2017 for an up-to-date overview of the key theories and references.

5 Well-being monism can, perhaps, be thought of as an instance of value monism, and the attractiveness of constitutive pluralism about well-being for many philosophers can be understood in light of this. Critics of value monism argue that common sense and empirical research suggests that we value lots of different things, and attempts to subsume all of our values and all of the objects of value under a single ‘supervalue’ fail to capture the complexity, conflict and incommensurability that characterises moral decision-making (Stocker 1990; Thomson 1997; Tiberius 2008). Constitutive value pluralism may be more or less monistic, depending on the extent to which it takes different bearers of value to share in some substantive property of ‘goodness’.

## Code Availability

There is no supporting code.

## References

[CR1] Alexandrova A (2017). A philosophy for the science of well-being.

[CR2] Angner E (2013). Is Empirical research relevant to philosophical conclusions?. Res Philosophica.

[CR3] Bishop M (2015). The good life.

[CR4] Brazier J (2010). Is the EQ–5D fit for purpose in mental health?. British Journal of Psychiatry.

[CR5] Brooks R, Group EQ (1996). EuroQol: The current state of play. Health Policy.

[CR6] Camfield L, Crivello G, Woodhead M (2009). Wellbeing research in developing countries: Reviewing the role of qualitative methods. Social Indicators Research.

[CR7] Campbell SM, Fletcher G (2016). The concept of well-being. The Routledge handbook of philosophy of well-being.

[CR8] Cartwright N (1999). The dappled world: A study of the boundaries of science.

[CR9] Chang H (2012). Is water H_2_O? Evidence, realism and pluralism.

[CR10] Chang H, Cartwright N, Curd M, Psillos S (2013). Measurement. The Routledge companion to philosophy of science.

[CR11] Clark AE, Flèche S, Layard R, Powdthavee N, Ward G (2018). The origins of happiness. The science of well-being over the life course.

[CR12] Crisp R (2006). Hedonism reconsidered. Philosophy and Phenomenological Research.

[CR13] Crisp R, Zalta EN (2017). Well-being. The stanford encyclopedia of philosophy.

[CR14] Crombie AC (1995). Commitments and styles of european scientific thinking. History of Science.

[CR15] Darwall S (2002). Welfare and rational care.

[CR16] Deaton A, Stone AA (2016). Understanding context effects for a measure of life evaluation: How responses matter. Oxford Economic Papers.

[CR17] Diener E, Lucas R, Schimmack U, Helliwell J (2009). Well-being for public policy.

[CR18] Dupré J (1993). The disorder of things: Metaphysical foundations of the disunity of science.

[CR19] Feldman F (2002). The good life: A defense of attitudinal hedonism. Philosophy and Phenomenological Research.

[CR20] Fletcher G (2013). A fresh start for the objective-list theory of well-being. Utilitas.

[CR21] Fletcher G (2019). Against contextualism about prudential discourse. The Philosophical Quarterly.

[CR22] Frijters, P., Clark, A. E., Krekel, C., & Layard, R. (2019). A happy choice: Wellbeing as the goal of government. IZA Institute of Labor Economics Discussion Paper 12720.

[CR23] Graham C, Macmillan P (2008). Happiness, Economics of. The new palgrave dictionary of economics.

[CR24] Griffin J (1988). Well-being: Its meaning, measurement and moral importance.

[CR25] Hawkins J (2019). Diversity of meaning and the value of a concept: Comments on Anna Alexandrova’s A philosophy for the science of well-being. Res Philosophica.

[CR26] Haybron DM (2008). The pursuit of unhappiness: The elusive psychology of well-being.

[CR27] Illari PM (2011). Mechanistic evidence: Disambiguating the Russo-Williamson thesis. International Studies in the Philosophy of Science.

[CR28] Kagan S (1994). Me and my life. Proceedings of the Aristotelian Society.

[CR29] Kellert SH, Longino HE, Kenneth Waters C (2006). Scientific pluralism. Minnesota studies in the philosophy of science,.

[CR30] Kneer, M. & Haybron D. M. (2020) Happiness and well-being: Is it all in your head? Evidence from the folk. Manuscript. https://www.researchgate.net/publication/337494445_Happiness_and_Well-Being_Is_It_All_in_Your_Head_Evidence_from_the_Folk.

[CR31] Layard R (2012). Why measure subjective well-being. Organisation for Economic Cooperation and Development OECD Observer.

[CR32] Layard, R. (2019, October). Evaluating well-being in the policy toolkit. *Paper presented to Putting Well-being Metrics into Policy Action*. Paris: OECD. Retrieved June 5, 2020, from https://www.oecd.org/statistics/putting-well-being-metrics-into-policy-action.htm.

[CR33] Layard, R., Clark, A., De Neve, J. E., Krekel, C., Fancourt, D., Hey, N., & O'Donnell, G. (2020). When to release the lockdown: A wellbeing framework for analysing costs and benefits. *London School of Economics Centre for Economic Performance Occasional Paper 49*.

[CR34] Longino HE (1990). Science as social knowledge: Values and objectivity in scientific inquiry.

[CR35] McClimans LM, Browne J (2011). Choosing a patient-reported outcome measure. Theoretical Medicine and Bioethics.

[CR36] Messick S (1995). Validity of psychological assessment: validation of inferences from persons' responses and performances as scientific inquiry into score meaning. American Psychologist.

[CR37] Mitchell, Polly (2018). *The Construction of Well-Being*. Ph.D. Thesis. University College London.

[CR38] Phillips J, De Freitas J, Mott C, Gruber J, Knobe J (2017). True happiness: The role of morality in the folk concept of happiness. Journal of Experimental Psychology: General.

[CR39] Phillips J, Misenheimer L, Knobe J (2011). The ordinary concept of happiness (and others like it). Emotion Review.

[CR40] Prinzing MM (2020). Positive psychology is value-laden—it's time to embrace it. The Journal of Positive Psychology.

[CR41] Richardson, J., McKie, J., & Bariola, E. (2011). *Review and critique of health related multi attribute utility instruments*. Monash University, Centre for Health Economics Research Paper 64.

[CR42] Roberts J, Dolan P (2004). To what extent do people prefer health states with higher values? A note on evidence from the EQ-5 D valuation set. Health Economics.

[CR43] Scanlon TM (1998). What we owe to each other.

[CR44] Sen AK (1985). Commodities and capabilities.

[CR45] Singh R, Alexandrova A (2020). Happiness economics as technocracy. Behavioural Public Policy.

[CR46] Steinberg J (2015). A man of good hope.

[CR47] Stiglitz JE, Sen A, Fitoussi J-P (2010). Mismeasuring our lives: Why GDP doesn't add up.

[CR48] Stocker M (1990). Plural and conflicting values.

[CR49] Thomson JJ (1997). The right and the good. Journal of Philosophy.

[CR50] Tiberius V (2008). The reflective life: Living wisely with our limits.

[CR51] Tiberius V (2011). How theories of well-being can help us help. Journal of Practical Ethics.

[CR52] Tiberius V, Plakias A, Doris JM, The Moral Psychology Research Group (2010). Well-being. The Moral Psychology Handbook.

[CR53] Wilson M (2006). Wandering significance: An essay on conceptual behavior.

[CR54] Wisløff T, Hagen G, Hamidi V, Movik E, Klemp M, Olsen JA (2014). Estimating QALY gains in applied studies: A review of cost-utility analyses published in 2010. PharmacoEconomics.

